# Endovascular management of aortoiliac artery occlusive disease with pseudo-stenosis of the external iliac artery

**DOI:** 10.1093/jscr/rjae078

**Published:** 2024-02-13

**Authors:** Hirokazu Matsushima, Tsunehiro Shintani, Hidenori Kita, Yuto Hasegawa

**Affiliations:** Department of Vascular Surgery, Shizuoka Red Cross Hospital, Shizuoka 420-0853, Japan; Department of Vascular Surgery, Shizuoka Red Cross Hospital, Shizuoka 420-0853, Japan; Department of Vascular Surgery, Shizuoka Red Cross Hospital, Shizuoka 420-0853, Japan; Department of Vascular Surgery, Shizuoka Red Cross Hospital, Shizuoka 420-0853, Japan

**Keywords:** aortoiliac artery occlusive disease, pseudo-stenosis, external iliac artery

## Abstract

In recent years, endovascular treatment has become the first-line revascularisation method for aortoiliac artery occlusive disease. Rarely, aortoiliac artery occlusive disease may be associated with stenosis of the external iliac artery (EIA) that suggested pseudo-stenosis. We describe a case of aortoiliac artery occlusive disease with EIA stenosis without calcification or atheroma. Stent grafts were inserted from the abdominal aorta to the bilateral common iliac arteries. Pre-operative computed tomography and intravascular ultrasound findings confirmed the absence of calcification or atheroma in both EIA, suggesting that the EIA had developed pseudo-stenosis. Following endovascular treatment, the EIA diameter recovered only with balloon dilation after inflow improvement. Consideration is necessary when placing an easy stent graft in the narrow EIA during endovascular treatment for aortoiliac artery occlusive disease with EIA stenosis to avoid a potential stent graft diameter mismatch.

## Introduction

Aortoiliac occlusive disease (AIOD) is a severe condition that often causes lower limb ischemia, necessitating surgical revascularization [1]. Occasionally, AIOD may be associated with external iliac artery (EIA) stenosis, resembling pseudo-stenosis. Therefore, we present a case of AIOD with EIA stenosis without calcification or atheroma, where the EIA diameter recovered only with balloon dilation following improved blood flow after endovascular AIOD treatment.

## Case report

**Figure 1:**
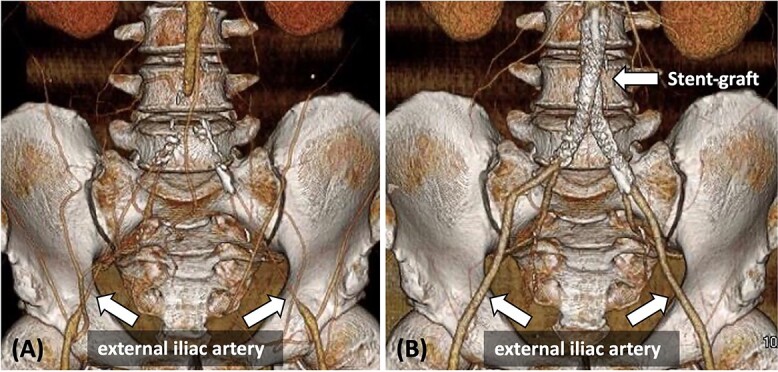
(A) Pre-operative contrast-enhanced CT reveals bilateral CIA occlusion from the abdominal aorta, with bilateral EIA stenosis, without calcification. (B) Contrast-enhanced CT 8 months post-surgery shows good bilateral EIA dilatation.

A 48-year-old man with a history of hypertension, dyslipidemia, and hyperuricemia presented with intermittent claudication for 50 m in both lower limbs. His body mass index (BMI) was 31.7 kg/m^2^. The patient’s right and left ankle-brachial index (ABI) values were 0.40 and 0.32, respectively. Contrast-enhanced computed tomography (CT) revealed complete bilateral common iliac artery (CIA) occlusion from the abdominal aorta at the inferior mesenteric artery (IMA) level (Fig. 1A). Furthermore, CT revealed bilateral EIA stenosis (right, 4.3 mm; left, 4.4 mm) without calcification or atheroma (Fig. 1A). He was diagnosed with AIOD with EIA stenosis, and was administered endovascular treatment because he was obese.

Initially, a 5-Fr sheath was inserted into the bilateral common femoral artery (CFA) under echo-graphic guidance and a 4-Fr sheath was inserted into the right brachial artery. A 0.035-inch Radifocus guidewire (TERUMO, Tokyo, Japan) and 4-Fr JR catheter were inserted from the right brachial artery to the abdominal aorta. The sheath was then switched to Parent Plus 45 (Medikit Co. Ltd., Tokyo, Japan). Angiography revealed that the abdominal aorta was occluded from the IMA level to the bilateral internal iliac artery bifurcations. Following unsuccessful attempts to insert the 0.035 inch Radifocus guidewire and 5-Fr catheter (HANACO MEDICAL Co., Ltd., Saitama, Japan) from the right CFA into the abdominal aorta, an ASAHI Gladius MG 14 PV ES guidewire (ASAHI INTECC CO., LTD., Aichi, Japan) and Fencer microcatheter (Medico’s Hirata Inc, Osaka, Japan) were successfully inserted. In the left CIA, inserting a guidewire similar to that used in the right CIA was challenging. Therefore, an ASAHI Halberd 0.014 guidewire (ASAHI INTECC CO., LTD., Aichi, Japan) was inserted up to the abdominal aorta.

**Figure 2:**
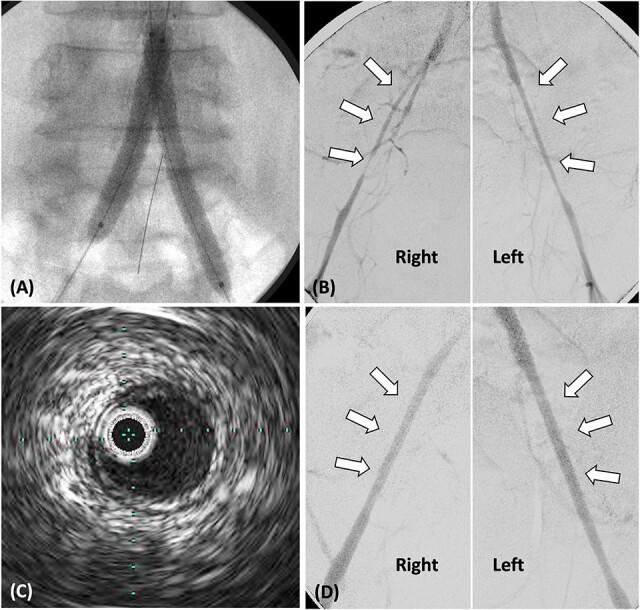
(A) Stent grafts placed from the abdominal aorta to bilateral CIA using the kissing stent technique. (B). Stenosis observed in both EIA, treated with plain balloon angioplasty only. (C) Post-balloon dilatation intravascular ultrasound reveals the absence of calcification or atheroma in both EIA. (D) Angiography post-balloon dilation reveals no EIA stenosis and calcification or atheroma.

Subsequently, stent grafts were placed from the abdominal aorta to the bilateral CIA. First, balloon dilatation was performed using 4.0 mm × 100 mm SHIDEN HP (Kaneka Medical Products, Osaka, Japan) from the abdominal aorta to bilateral CIA. The sheath inserted in the bilateral CFA was switched to a 7-Fr long sheath (25 cm), whereas the guidewire was changed to a 0.035-inch Radifocus guidewire. Second, Gore Viabahn VBX stent-grafts (W. L. Gore and Associates Inc., Flagstaff, AZ, USA) were inserted from the abdominal aorta at the IMA level to both CIAs (right: 8 × 59 mm and 8 × 59 mm; left: 8 × 59 mm and 7 mm × 59 mm). Finally, both VBX stent-grafts were individually dilated with a 10 mm × 40 mm Mustang balloon (Boston Scientific, Marlborough, MA, USA) and then molded using a kissing balloon technique with an 8-mm balloon [2] (Fig. 2A). To prevent IMA occlusion, IMA was shielded with a 4.0 mm × 40 mm Sterling balloon (Boston Scientific, Marlborough, MA, USA) during the procedure. Express SD stents (4 × 19 mm and 4 × 19 mm; Boston Scientific, Marlborough, MA, USA) were eventually placed at the origin of the IMA.

Following stent-graft placement, angiography revealed severe EIA stenosis and delayed blood flow (Fig. 2B). Both EIA lengths were carefully dilated using a 5.0 × 100 mm SHIDEN HP balloon. After balloon dilatation, delayed blood flow resolved. Intravascular ultrasound (IVUS) finding post-balloon dilatation confirmed the absence of calcification or atheroma in both EIA, suggesting that EIA was pseudo-stenosis and no additional stents were inserted (Fig. 2C and D).

On post-operative day 2, the patient exhibited an ABI value of 0.99 in both lower limbs, recovered well, and was discharged. Eight months post-surgery, contrast-enhanced CT revealed no stenosis, and both EIA diameters improved (right: 8.3 mm, left: 7.5 mm) (Fig. 1B).

## Discussion

AIOD results in symptoms including claudication, decreased femoral pulses, and impotence [1]. It causes lower limb ischemia, leading to severe chronic lower limb ischemia in 30–40% of patients [1]. In cases of AIOD wherein the obstruction has not spread to the renal artery, there are no clear criteria for open surgery or endovascular treatment [3]. Recent reports show favorable outcomes with endovascular treatment for AIOD [4]. Aortobifemoral bypass in obese AIOD patients has been reported to have longer operative time and length of stay than in non-obese patients [5]. In this case, open surgery was recommended due to the patient’s young age, but because the patient was obese with a BMI of 31.7 kg/m^2^, a less invasive endovascular treatment was performed in accordance with the patient’s wishes.

Pseudo-stenosis of the EIA following AIOD surgical bypass has been reported [6], but endovascular treatment reports are lacking. Surgeons find it challenging to distinguish between true and pseudo-stenosis when AIOD is complicated by EIA stenosis. Elongated stenotic lesions without calcification or a bird-beak-like appearance at the distal EIA on CT, as in this case, indicate pseudo-stenosis [6]. Furthermore, the endovascular treatment enables determining whether the EIA lumen is associated with an atheroma through IVUS. If EIA stenosis is pseudo-stenosis, post-operative EIA is likely to dilate and recover vessel diameter due to improved blood flow from the aorta and iliac arteries. Stent placement solely due to narrow EIA diameter carries a high risk of diameter mismatch and potential stent migration after the post-operative EIA dilatation.

In conclusion, when performing endovascular treatment for AIOD with EIA stenosis, careful evaluation of EIA therapeutic intervention is important. If the EIA lacks calcification or atheroma on pre-operative CT or IVUS, pseudo-stenosis should be suspected. Therefore, restrict treatment to percutaneous transluminal angioplasty while avoiding easy stent placement.
